# Renal Protective Effect of Beluga Lentil Pretreatment for Ischemia-Reperfusion Injury

**DOI:** 10.1155/2021/6890679

**Published:** 2021-01-31

**Authors:** Syng-ook Lee, So Young Chun, EunHye Lee, Bomi Kim, BoHyun Yoon, Haejung Gil, Dae Hwan Kim, Byung Ik Jang, Dong Woo Lee, Eun Sang Yoo, Dong Jin Park, Jun Nyung Lee, Man-Hoon Han, Bum Soo Kim, Phil Hyun Song, Tae Gyun Kwon, Yun-Sok Ha

**Affiliations:** ^1^Department of Food Science and Technology, Keimyung University, Daegu 42601, Republic of Korea; ^2^BioMedical Research Institute, Kyungpook National University Hospital, Daegu 41940, Republic of Korea; ^3^Department of Laboratory Animal Research Support Team, Yeungnam University Hospital, Daegu 42415, Republic of Korea; ^4^Department of Internal Medicine, Yeungnam University College of Medicine, Daegu 42415, Republic of Korea; ^5^Department of Urology, School of Medicine, Kyungpook National University, Daegu 41566, Republic of Korea; ^6^Department of Pathology, School of Medicine, Kyungpook National University, Daegu 41566, Republic of Korea; ^7^Department of Urology, Yeungnam University College of Medicine, Daegu 42415, Republic of Korea

## Abstract

**Materials and Methods:**

Mice were divided into four groups: normal, untreated, low- (2 mg), and high-dose (8 mg) beluga lentil treatment groups. Beluga lentil was orally administered for 2 weeks, followed by bilateral renal ischemia for 20 min and reperfusion for 30 min. Blood samples and kidney tissues were collected and analyzed to investigate renal function, histopathology, epithelial and endothelial cell damage, apoptosis, oxidative stress, and inflammatory responses.

**Results:**

The pretreated groups maintained renal function, with significantly lower blood urea nitrogen (BUN) and creatinine levels, compared with the other groups. The histopathological analysis showed reduced proximal tubule injury and decreased injury-related molecule (kidney injury molecule 1 (KIM-1) and neutrophil gelatinase-associated lipocalin (NGAL)) secretion in the pretreated groups compared with the other groups. Terminal deoxynucleotidyl transferase dUTP nick-end labeling- (TUNEL-) positive cells and the secretion of apoptosis-related molecules (Fas and caspase 3) were significantly reduced in the pretreated groups compared with the other groups. The pretreated groups showed positive microvessel-associated gene (cluster of differentiation (CD31)) expression and negative adhesion molecule (intracellular adhesion molecule 1 (ICAM-1)) expression. An antioxidant effect was observed in the pretreatment groups, with reduced malonaldehyde (MDA) expression and increased antioxidant enzyme (superoxide dismutase (SOD), catalase (CAT), glutathione (GSH), and glutathione peroxidase (GPx)) secretion. In the pretreated groups, F4/80+ macrophages and CD4+ T cell infiltration were inhibited and proinflammatory cytokine (interleukin- (IL-) 1*β*, IL-6, and tumor necrosis factor- (TNF-) *α*) levels decreased; however, the levels of anti-inflammatory cytokines (transforming growth factor- (TGF-) *β*, IL-10, and IL-22) increased.

**Conclusions:**

Beluga lentil pretreatment demonstrated protective effects against I/R-induced renal damage, via antiapoptotic, anti-inflammatory, and antioxidant activities.

## 1. Introduction

Ischemia/reperfusion (I/R) injury, during partial nephrectomy or renal transplantation, causes acute renal injury [1, 2] and may result in the irreversible deterioration of renal function [3]. Ischemia triggers apoptosis in renal tubular epithelial cells, which amplifies the inflammatory responses of interstitial cells. Reperfusion induces microvascular damage, which promotes inflammatory cell migration, through adhesion factor expression on the surface of endothelial cells [4–8]. I/R injury also causes oxidative stress, increasing reactive oxygen species (ROS) and decreasing antioxidant enzyme activity [9], resulting in the increased production of proinflammatory factors [10], caspase pathway activation, and increased apoptotic cell death, which eventually cause the loss of renal function [11]. To prevent I/R-induced renal injury, the use of antioxidant agents, with anti-inflammatory and antiapoptotic functions, has been proposed [10–14].

Lentil cultivars (green, red, French, or beluga) contain various bioactive compounds, especially antioxidants [15]. Their total polyphenol and flavonoid contents range from 27.30–30.30 mg (tannic acid equivalents)/g to 13.14–16.29 mg (quercetin equivalents)/g, respectively [16]. Beluga lentils have been demonstrated to have significantly high polyphenol contents and ROS scavenging effects [16]. Our team reported the antioxidant effects of lentils, using an in vitro liver cell line experiment [16]. Beluga lentils demonstrated significant protective effects against alcohol-induced cytotoxicity in AML-12 cells compared with other lentil cultivars. The anti-inflammatory effects of beluga lentils were also observed in lipopolysaccharide-treated RAW264.7 cells [17]. Beluga lentil treatment significantly decreased nitric oxide (NO) production and inducible NO synthase (iNOS) expression, through the upregulation of the nuclear factor E2-related factor 2- (Nrf2-) mediated heme oxygenase-1 (HO-1) pathway. These in vitro experiments suggested the antioxidative and anti-inflammatory effects of beluga lentils.

To expand the applications of beluga lentils, we applied them to a renal I/R injury mouse model and evaluated the renal protective effects. For this experiment, beluga lentils were administered for 2 weeks, as a pretreatment, followed by ischemia for 20 min and reperfusion for 30 min. The renal protective effects of beluga lentil pretreatment were verified by analyzing renal function, histopathology, epithelial and endothelial cell damage, apoptosis, oxidative stress, and inflammatory responses. We hypothesized that pretreatment with beluga lentils would prevent I/R-induced renal injury, via antioxidant, anti-inflammatory, and antiapoptotic activities.

## 2. Materials and Methods

### 2.1. Animal Groups and Treatment Conditions

All procedures were performed using an animal protocol that has been approved by the Yeungnam University Institutional Animal Care and Use Committee (AEC2019-003). Mice (ICR, 8 weeks old, male, 23–25 g, Orient, Seongnam, Korea) were randomly divided into the following 4 groups (*n* = 7 per group): (1) Normal, normal control group; (2) Untreated, saline-treated group; (3) Low, low-dose (2 mg/100 *μ*L saline/mouse), 14-day orally administered beluga lentil pretreatment group; and (4) High, high-dose (8 mg/100 *μ*L saline/mouse), 14-day orally administered beluga lentil pretreatment group. Beluga lentils were provided by Prof. Syng-Ook Lee (Keimyung University, Daegu, Korea), and the extract preparation and bioactive compound analysis were reported in a previous study [16]. After treatment, the animals were placed in a prone position, under anesthesia, and a dorsal incision was made [1]. The renal artery and veins for both kidneys were occluded with a vascular clamp for 20 min, followed by reperfusion for 30 min, according to a previously described protocol [18, 19]. Blood was collected by cardiac puncture, and the kidneys were extracted. The kidneys were washed with phosphate-buffered saline; one kidney was used for RNA and protein extraction, whereas the other kidney was used for histological analysis.

### 2.2. Histopathological and Immunohistochemical (IHC) Analyses

Histopathologic examinations were performed using hematoxylin and eosin (H&E) staining, and injuries were evaluated based on the following factors: the presence of tubule cells falling into the lumen, nuclear loss in exfoliated cells, luminal debris, collapsed luminal space, and immune cell infiltration. The scoring was achieved by a pathological specialist: score 0, no tubular injury; score 1, <10% of tubules injured; score 2, 10–25% of tubules injured; score 3, 25–50% of tubules injured; score 4, 50–74% of tubules injured; and score 5, >75% of tubules injured. For IHC analysis, the kidney was fixed with 4% paraformaldehyde, and paraffin-embedded samples were cut into 5 *μ*m sections. H&E and IHC staining were performed, following routine processes. Primary antibodies against immune cell (F4/80 and cluster of differentiation 8 (CD8), Abcam, Cambridge, UK) and endothelial cell (CD31 and intracellular adhesion molecule 1 (ICAM-1), Abcam) markers were applied to sections, for 24 h at 4°C (dilution 1 : 200), followed by the secondary antibody (Alexa Fluor 594, Life Technology, Waltham, MA, USA), for 1 h at room temperature, and 4′,6-diamidino-2-phenylindole (DAPI) was used to stain nuclei.

### 2.3. Protein Assay

For renal function analysis, serum was separated, without an anticoagulant, and serum creatinine and blood urea nitrogen (BUN) concentrations were detected using a Creatinine Colorimetric Assay Kit and a QuantiChrom Urea Assay Kit (BioAssay Systems LLC, Hayward, CA, USA), respectively. To assess renal tubule/vessel injury, oxidative stress, antioxidant enzymes, and apoptosis, kidney tissue was homogenized with each respective buffer. To analyze renal tubule epithelial cell injury, kidney injury molecule-1 (KIM-1) and neutrophil gelatinase-associated lipocalin (NGAL) concentrations were assessed using enzyme-linked immunosorbent assay (ELISA) (USCN Life Science Inc., Wuhan, China). To analyze apoptosis, Fas and caspase 3 concentrations were measured using respective ELISA kits (Abcam). To evaluate oxidative stress, malonaldehyde (MDA) levels were measured using an MDA assay kit (Nanjing Jiancheng Bioengineering Research Institute, Nanjing, China). Antioxidant enzymes, including superoxide dismutase (SOD), catalase (CAT), glutathione (GSH), and glutathione peroxidase (GPx), were measured using a total SOD assay kit (Nanjing Jiancheng Bioengineering Research Institute), a CAT assay kit (Nanjing Jiancheng Bioengineering Research Institute), a GSH fluorometric assay kit, and a GPx Assay Kit (BioVision Inc.), respectively. All kits were used according to the manufacturer's instructions.

### 2.4. Gene Expression Analysis

Total RNA was extracted with the TRIzol Reagent, and cDNA was synthesized from 20 *μ*g total RNA, using a cDNA synthesis kit (Invitrogen, Waltham, MA, USA). The real-time PCR conditions were as follows: 95°C for 10 min, followed by 40 cycles of 95°C for 10 sec, 60°C for 50 sec, and 72°C for 20 sec. Gene amplification was detected using SYBR green, and the 2^−ΔΔCt^ method was used to analyze expression. Experiments were performed in triplicate, using the following primer sequences: interleukin- (IL-) 1*β*, 5′-gcccatcctctgagactcat-3′ and 5′-aggccacaggtattttgtcg-3′; IL-6, 5′-agttgccttcttgggactga-3′ and 5′-tccacgatttcccagagaac-3′; tumor necrosis factor- (TNF-) *α*, 5′-agcccccagtctgtatcctt-3′ and 5′-ctccctttgcagaactcagg-3′; transforming growth factor- (TGF-) *β*, 5′-tggttgtagagggcaaggac-3′ and 5′-ttgcttcagctccacagaga-3′; IL-10, 5′-acctggtagaagtgatgccc-3′ and 5′-agggtcttcagcttctcacc-3′; IL-22, 5′-tccaacttccagcagccata-3′ and 5′-tagcactgactcctcggaac-3′; and glyceraldehyde 3′-phosphate dehydrogenase (GAPDH), 5′-tgtgtccgtcgtggatctga-3′ and 5′-cctgcttcaccaccttcttga-3′.

### 2.5. TUNEL Assay

To assess apoptosis, TdT-mediated dUTP nick-end labeling (TUNEL) assay was performed using an apoptosis detection kit (Chemicon, Bedford, MA, USA) following the manufacturer's instructions. Briefly, deparaffinized and rehydrated slides were digested with 20 *μ*g/mL proteinase K, at 37°C for 15 h, to remove proteins, and treated with 3.0% hydrogen peroxide, to quench endogenous peroxidase. The slides were immersed in 1x TdT equilibration buffer, and a working strength of the TdT enzyme was added for 1 h at 37°C. An antidigoxigenin conjugate was applied to the 3′-OH DNA terminus for 30 min, and the color was developed using a peroxidase substrate for 3 min. After DAPI treatment, the slides were mounted. TUNEL-positive nuclei were counted in all visual fields in each tissue sample, under 200x magnification.

### 2.6. Statistical Analysis

All values are expressed as the mean ± standard deviation. Significant differences for the I/R renal injury group and the beluga lentil-I/R pretreatment groups were evaluated using an analysis of variance, followed by Tukey's post hoc test, in SPSS (Statistical Package for the Social Sciences v. 9.0; Chicago, IL, USA). *p* values < 0.05 were considered significant.

## 3. Results

### 3.1. Effects of Beluga Lentil Pretreatment on Renal Function

**Figure 1:**
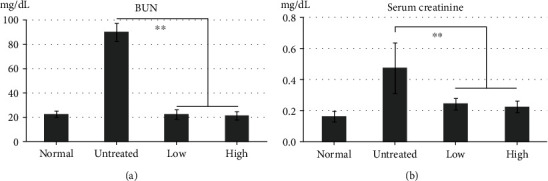
Effects of beluga lentil pretreatment on renal function after ischemia/reperfusion injury. BUN (a) and serum creatinine (b) levels were significantly reduced in the pretreatment groups compared with the untreated group. Normal: control group; untreated: saline-treated group; low: 2 mg beluga lentil pretreatment group; high: 8 mg beluga lentil pretreatment group. Beluga lentil was orally administered for 14 days, and then ischemia was performed for 20 min and reperfusion was performed for 30 min (^∗∗^*p* < 0.01). BUN: blood urea nitrogen.

The pretreated groups both showed significant protective effects against I/R injury (Figure 1). The mean BUN and serum creatinine concentrations in the untreated group were 89.92 ± 7.29 mg/dL and 0.48 ± 0.16 mg/dL, respectively. The pretreated groups showed significantly reduced BUN (low: 22.6 ± 3.67 mg/dL; high: 21.6 ± 3.17 mg/dL) and serum creatinine (low: 0.25 ± 0.04 mg/dL; high: 0.23 ± 0.04 mg/dL) concentrations compared with those in the untreated group (*p* < 0.01), and no significant differences were observed between the two pretreatment groups. These results indicated that pretreatment with beluga lentils can protect against acute renal functional deterioration.

### 3.2. Effects of Beluga Lentil Pretreatment on Tubular Epithelial Cell Injury and Apoptosis

**Figure 2:**
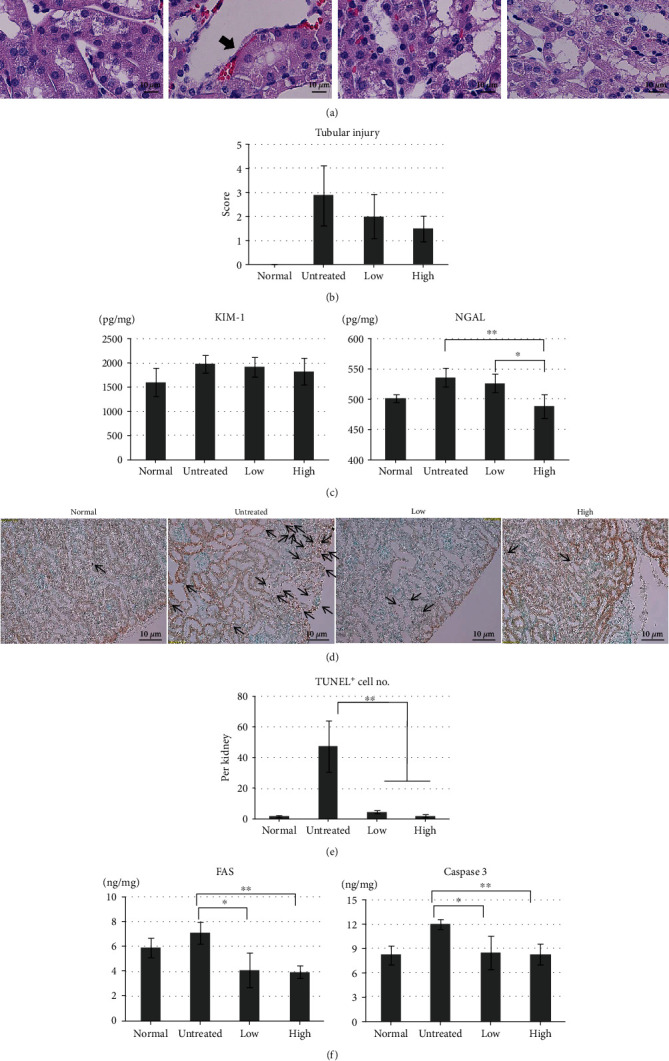
Effects of beluga lentil pretreatment on renal tubular epithelial cells after ischemia/reperfusion injury. (a) Histopathology with hematoxylin and eosin (H&E) stain. Arrow indicates damaged tubular epithelial cells in the outer medulla. (b) Quantification of tubular injury, as a percentage per unit area. (c) Quantification of KIM-1 and NGAL (tubular epithelial cell injury markers) concentrations, using ELISA. NGAL is significantly reduced in the lentil pretreatment group. (d) TUNEL assay to identify apoptotic cells. Arrow indicates apoptotic fragmented chromosomal DNA. (e) Quantification of TUNEL-positive cells in each kidney. (f) Quantification of Fas and caspase 3 (apoptotic cell injury markers) concentrations, using ELISA. Fas is significantly reduced in the beluga lentil pretreatment group. Normal: control group; untreated: saline-treated group; low: 2 mg beluga lentil pretreatment group; high: 8 mg beluga lentil pretreatment group. Beluga lentil was orally administered for 14 days, and then ischemia was performed for 20 min and reperfusion was performed for 30 min. Scale bar: 10 *μ*m (^∗∗^*p* < 0.01 and ^∗^*p* < 0.05). KIM-1: kidney injury molecule-1; NGAL: neutrophil gelatinase-associated lipocalin; TUNEL: terminal deoxynucleotidyl transferase dUTP nick-end labeling.

Tubular injury, especially the atrophy of epithelial cells, was frequently observed in the outer kidney medulla, and exfoliated cell nuclear loss, luminal debris on the renal tubule, collapsed luminal space, and interstitial neutrophil infiltrates were occasionally identified in the kidneys of the untreated group (Figure 2(a)). In contrast, the kidneys from the pretreated groups showed nearly normal cellular morphologies, and tubular injury was less frequently observed in the high-dose group than in the low-dose group. When the injury was expressed as a percentage per unit area, the injury score was reduced in the pretreated groups (low: 2.0 ± 0.93; high: 1.5 ± 0.53) compared with that in the untreated group (2.87 ± 1.25) (Figure 2(b)). When examining KIM-1 and NGAL secretion (Figure 2(c)), the ELISA results demonstrated that KIM-1 contents decreased in the pretreated groups (low: 1, 922.83 ± 199.42 pg/mg; high: 1,827.83 ± 278.26 pg/mg) compared with that in the untreated group (1,977.83 ± 184.49 pg/mg). NGAL expression was significantly reduced in the high-dose group (489.34 ± 19.95 pg/mg) compared with those in the low-dose (527.36 ± 15.19 pg/mL) and untreated (537.05 ± 15.70 pg/mg) groups (*p* < 0.01).

Whether tubular epithelial cell injury caused apoptosis was then analyzed. Cellular apoptosis was assessed by detecting fragmented chromosomal DNA, using the TUNEL assay (Figure 2(d)). TUNEL-positive cells were rarely observed in the pretreated groups (low: 5.00 ± 1.22; high: 2.25 ± 1.09). In contrast, the untreated group showed relatively increased numbers of TUNEL-positive cells in the outer medulla region (47.50 ± 16.77) (*p* < 0.01), showing apoptotic bodies that extruded into the tubular lumen (Figure 2(e)). When the secretion of apoptosis-related molecules (Fas and caspase 3) was analyzed by ELISA (Figure 2(f)), Fas expression was decreased in the pretreated groups (low: 4.10 ± 1.39 ng/mg; high: 3.96 ± 0.55 ng/mg) compared with that in the untreated group (7.10 ± 0.87 ng/mg), and caspase 3 expression showed similar results (low: 8.44 ± 2.05 ng/mg; high: 8.25 ± 1.28 ng/mg; and untreated: 11.99 ± 0.63 ng/mg) (*p* < 0.05). These results indicated that beluga lentil pretreatment prevented tubular epithelial cell apoptosis induced by I/R.

### 3.3. Effects of Beluga Lentil Pretreatment on Endothelial Cell Injury

**Figure 3:**
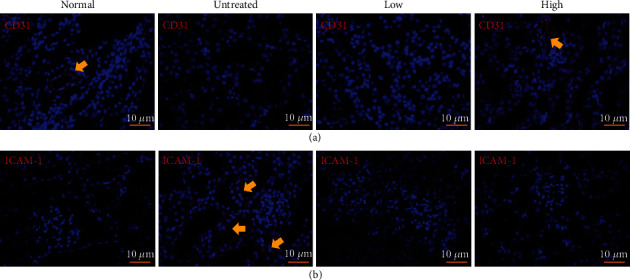
Effects of beluga lentil pretreatment on renal endothelial cells after ischemia/reperfusion injury. Immunohistochemical analysis examining CD31 (a) and ICAM-1 to detect endothelial cell damage (b). Arrow indicates an existing microvessel and damaged endothelial cell. Normal: control group; untreated: saline-treated group; low: 2 mg beluga lentil pretreatment group; high: 8 mg beluga lentil pretreatment group. Beluga lentil was orally administered for 14 days, and then ischemia was performed for 20 min and reperfusion was performed for 30 min. CD31: cluster of differentiation 31; ICAM-1: intercellular adhesion molecule 1.

The effects of beluga lentil pretreatment on endothelial cells were verified by IHC, using a CD31 antibody (Figure 3(a)). In the normal group, CD31-positive blood vessels were identified, but CD31-positive cells were not identified in the untreated group, indicating I/R-induced vascular disruption. The high-dose pretreatment group showed CD31-positive cells in the peritubular capillaries, indicating a vessel-protective effect of high-dose beluga lentil pretreatment. Endothelial cells were examined by detecting ICAM-1, an adhesion molecule, and positive cells were more frequently identified in the tubular region of the untreated group (46.4 ± 41.7 cells/slide) compared with the pretreated groups (low: 5.77 ± 1.01 cells/slide; high: 3.72 ± 1.05 cells/slide) (Figure 3(b)). These results indicated that beluga lentil pretreatment preserved capillaries and inhibited adhesion molecule activation on the endothelial cell surface.

### 3.4. Effects of Beluga Lentil Pretreatment on Oxidative Stress

**Figure 4:**
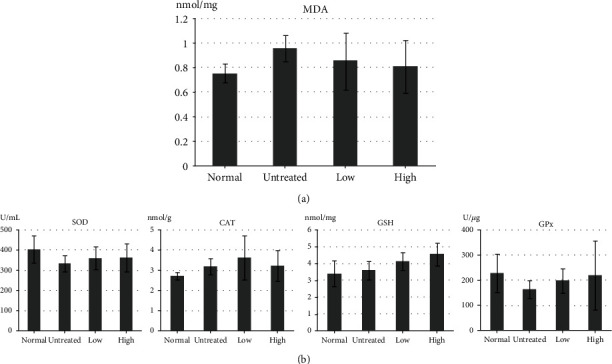
Effects of beluga lentil pretreatment on oxidative stress after ischemia/reperfusion injury. Quantification of MDA (a) and antioxidant enzyme (b) concentrations, using ELISA. Normal: control group; untreated: saline-treated group; low: 2 mg beluga lentil pretreatment group; high: 8 mg beluga lentil pretreatment group. Beluga lentil orally administered for 14 days, and then ischemia was performed 20 min and reperfusion was performed for 30 min. MDA: malondialdehyde; SOD: superoxide dismutase; CAT; catalase; GSH: glutathione; GPx: glutathione peroxidase.

To evaluate the antioxidant effects of beluga lentil pretreatment, MDA, SOD, CAT, GSH, and GPx levels were detected by ELISA (Figure 4). MDA levels decreased in the pretreated groups (low: 0.85 ± 0.23 nmol/mg; high: 0.81 ± 0.22 nmol/mg) compared with that in the untreated group (0.96 ± 0.11 nmol/mg) (*p* > 0.05) (Figure 4(a)). However, the antioxidant enzyme activities increased in the pretreated groups (Figure 4(b)) compared with those in the untreated group (*p* > 0.05), for SOD (low: 360.21 ± 56.42 U/mL; high: 362.70 ± 70.32 U/mL; and untreated: 333.52 ± 41.58 U/mL), CAT (low: 3.64 ± 1.09 nmol/g; high: 3.21 ± 0.76 nmol/g; and untreated: 3.18 ± 0.40 nmol/g), GSH (low: 4.13 ± 0.53 nmol/mg; high: 4.56 ± 0.69 nmol/mg; and untreated: 3.59 ± 0.53 nmol/mg), and GPx (low: 198.89 ± 48.43 U/*μ*g; high: 219.61 ± 138.08 U/*μ*g; and untreated: 165.14 ± 34.73 U/*μ*g). Although these differences were not statistically significant, these results suggested that beluga lentil pretreatment prevented I/R-induced renal oxidative stress by enhancing the antioxidant potency in kidneys.

### 3.5. Effects of Beluga Lentil Pretreatment on Immune Cell Infiltration, Cytokines, and Inflammation

**Figure 5:**
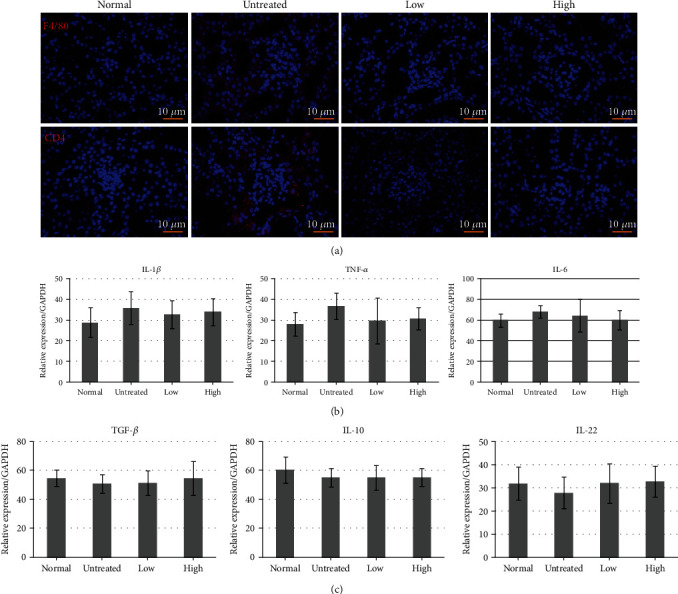
Effects of beluga lentil pretreatment on inflammatory responses after ischemia/reperfusion injury. (a) Immunohistochemical analysis examining F4/80 and CD4 to detect immune cell infiltration. (b) Detection of proinflammatory cytokines IL-1*β*, TNF-*α*, and IL-6 mRNA expression. (c) Detection of anti-inflammatory cytokines TGF-*β*, IL-10, and IL-22 mRNA expression. Normal: control group; untreated: saline-treated group; low: 2 mg beluga lentil pretreatment group; high: 8 mg beluga lentil pretreatment group. Beluga lentil was orally administered for 14 days, and then ischemia was performed for 20 min and reperfusion was performed for 30 min. F4/80: macrophage marker; CD4: cluster of differentiation 4; IL-1*β*: interleukin 1 beta; IL-6: interleukin 6; TNF-*α*: tumor necrosis factor-alpha; IL-10: interleukin 10; TGF-*β*1: transforming growth factor beta 1.

Macrophage (F4/80+) and T cell (CD4+) infiltration and proinflammatory cytokine (IL-1*β*, IL-6, and TNF-*α*) release were analyzed to evaluate apoptosis-induced inflammation. Fewer F4/80+ and CD4+ infiltrating immune cells were observed in the pretreated groups than in the untreated group, as assessed by IHC analysis (Figure 5(a)). The real-time PCR analysis showed that the renal mRNA levels of proinflammatory cytokine (IL-1*β*, TNF-*α*, and IL 6) levels decreased in the pretreated group compared with those in the untreated group (Figure 5(b)). However, the mRNA levels of anti-inflammatory cytokines (TGF-*β* and IL-22) increased in the pretreated groups compared with the untreated group, except for IL-10 (Figure 5(c)). These results indicated that I/R-induced inflammatory injury in the kidneys may be prevented by beluga lentil pretreatment due to anti-inflammatory effects, although IL-10 was not affected.

## 4. Discussion

Renal injury induced by I/R is traditionally characterized by rapidly decreasing kidney function [20]. Renal function can be estimated using BUN and serum creatinine values, which are nitrogenous metabolic end-products that indicate glomerular filter function [21]. The untreated group showed significantly increased BUN (89.92 ± 7.29 mg/dL) and creatinine (0.48 ± 0.16 mg/dL) values, indicating functional kidney failure induced by I/R. However, the beluga lentil pretreated groups showed significantly reduced BUN (low: 22.6 ± 3.67 mg/dL; high: 21.6 ± 3.17 mg/dL) and serum creatinine (low: 0.25 ± 0.04 mg/dL; high: 0.23 ± 0.04 mg/dL) values (*p* < 0.01) compared with those in the untreated group. These values are similar to those observed in normal mice (male, 6 wks; BUN: 22.68 ± 3.05 mg/dL; creatinine: 0.25 ± 0.06 mg/dL) [22]. The significantly decreased BUN and serum creatinine values observed in the pretreated groups relative to the untreated group indicated that beluga lentil pretreatment exerted protective effects on renal function against I/R-induced renal injury.

Decreased renal function indicates glomerular filtration problems caused by the back leak of the glomerular filtrate across the tubular epithelium, which represents the region that is the most severely injured by I/R [23]. I/R resulted in exfoliated cell nuclear loss, luminal debris on the renal tubule, and collapsed luminal spaces in tubular epithelial cells. The pretreated groups showed reduced histopathologic tubular injury in a dose-dependent manner. The observed histopathologic tubule injury was confirmed by assessing molecular markers, KIM-1 and NGAL [24]. KIM-1 is a phosphatidylserine receptor that acts as an apoptotic cell recognition molecule, transferring an injured cell to the lysosome for apoptotic/necrotic cell phagocytosis, apoptotic debris clearance, and proinflammatory response limitation [25]. NGAL is a small siderophoric protein that binds to gelatinases derived from human neutrophils. NGAL is rarely expressed in normal kidneys, but acute, ischemic, or toxic kidney damages result in NGAL secretion by the epithelial cells in the proximal/distal tubules, increasing the NGAL concentrations in urine and blood; thus, NGAL can be used as a novel early biomarker for I/R-induced acute renal failure [26]. The pretreated groups showed reduced KIM-1 and NGAL secretion, and the high-dose pretreatment group showed a significant reduction in NGAL secretion compared with the untreated group. The observed reductions in tubular injury and KIM-1 and NGAL protein secretion indicated that beluga lentil pretreatment exerted protective effects against tubular epithelial cell damage induced by I/R.

When I/R injury is severe, damaged cells are cleared through the apoptotic pathway, which is a pathophysiological cell-death mechanism [27]. Apoptosis-specific DNA damage can be detected through the TUNEL assay. TUNEL-positive cells were frequently observed in the untreated group, whereas the beluga lentil pretreated groups showed significantly reduced TUNEL-positive cell numbers, in a dose-dependent manner. This histological evidence of apoptosis was confirmed by examining Fas and caspase 3 expression. Fas is a ligand that binds to the cell-death receptor and is initially observed during programmed cell death [28]. Caspase 3 is an intracellular execution enzyme of the apoptotic cell-death pathway, resulting in apoptotic body formation [29]. Fas and caspase 3 protein syntheses were significantly decreased in the pretreated group compared with those in the untreated group, confirming that beluga lentil pretreatment can inhibit apoptotic signal transduction following I/R injury.

I/R injury also induces the loss of renal microvessels [30], resulting in pathohistological changes in endothelial cells [20]. Renal microvessel damage was detected using an antibody against CD31, an endothelial cell marker [30]. CD31 expression was not detected in the untreated group, indicating the loss of renal microvessels. However, the high-dose pretreated group showed positive CD31 expression, with a similar vascular density as observed in the normal group, which indicated that beluga lentil pretreatment, at high doses, exerted a protective effect on endothelial cell preservation. Damaged endothelial cells express endothelium-leukocyte adhesion molecules, such as ICAM-1 [31]. Increased adhesion molecules can lead to leukocyte activation, capillary obstruction, cytokine production, and proinflammatory responses [32]. The untreated group showed positive ICAM-1 expression, indicating enhanced adhesion molecule expression induced by endothelial cell damage. However, the high-dose pretreated group showed negative ICAM-1 expression, indicating the protective effects of beluga lentil pretreatment for the maintenance of endothelial cells, both functionally and physiologically. The maintained vascular structures can provide consistent blood flow, delivering oxygen and nutrients to tissues and cells damaged by I/R, assisting in the functional and histological recovery of the kidney.

Reperfusion restores the oxygen supply to damaged cells, which can trigger the expression of oxidative stress-related enzymes, which convert oxygen into ROS [33]. ROS are unstable and highly reactive products that generate free radicals, which cause DNA damage, apoptosis, and necrosis, through the change in cellular proteins, lipids, and nucleic acids. Free radicals damage unsaturated fatty acids in the cell membrane, resulting in lipid peroxidation [34]. Lipid peroxide is then degraded into MDA, which reduces antioxidant enzyme activities, such as SOD, GPx, GSH, and CAT [35]. SOD catalyzes the dismutation of superoxide into oxygen and hydrogen peroxide, which is further degraded by CAT. GPx is a key antioxidant enzyme that eliminates peroxide, using GSH. GSH can detoxify various oxidative products, in combination with GPx. MDA and antioxidant enzyme levels demonstrated an inverse relationship in the ELISA analysis. In the pretreated groups, MDA expression was relatively low, but the antioxidant enzymes were highly expressed compared with their respective levels in the untreated group. These results indicated that beluga lentil pretreatment can reduce I/R-induced renal damage by blocking the oxidative stress pathway, through the inhibition of membrane lipid peroxidation and the activation of antioxidant enzymes.

Reperfusion also triggers vascular endothelial cell activation, resulting in ICAM-1 expression on the cell surface. The expressed ICAM-1 connects with neutrophils through lymphocyte function-associated antigen-1 (LFA-1), which results in the attachment of neutrophils to endothelial cells, neutrophil infiltration, and the secretion of inflammatory cytokines by neutrophils [36]. After neutrophils appear in a damaged region, the infiltration of F4/80+ macrophages occurs. Macrophages differentiate into proinflammatory M1 and anti-inflammatory M2 phenotypes, depending on time and the surrounding environment. M1 macrophages secrete proinflammatory cytokines (IL-1*β*, TNF-*α*, and IL-6) which are associated with I/R-induced renal damage, whereas M2 macrophages secrete anti-inflammatory cytokines (TGF-*β*, IL-10, and IL-22) [37, 38]. Macrophages also stimulate CD4+ T cell activation. Activated T cells synthesize interferon- (IFN-) *γ*, which amplifies the immune response through M1 macrophage activation [39]. In this study, the beluga lentil pretreated groups showed inhibitory effects against F4/80+ macrophage and CD4+ T cell infiltration into the renal cortex region, resulting in the decreased expression of IL-1*β*, IL-6, and TNF-*α* mRNA and the enhanced expression of TGF-*β*, IL-10, and IL-22 mRNA. These results indicated that beluga lentil pretreatment can reduce the inflammatory response by inhibiting immune cell infiltration and reducing inflammatory cytokine expression.

## 5. Conclusions

In summary, the beluga lentil pretreatment groups showed reduced proximal tubule injury, decreased injury-related molecule secretion, reduced TUNEL-positive cells, decreased apoptosis-related molecule secretion, positive microvessel expression, negative adhesion marker expression, an antioxidant effect, and inhibited inflammatory responses. Therefore, beluga lentil pretreatment exerted protective effects against I/R-induced renal damage, via antiapoptotic, anti-inflammatory and antioxidative activities. Based on the results of this study, renal function can be preserved by using beluga lentil treatments in clinical situations associated with I/R injury, such as partial nephrectomy.

## Data Availability

Data is available on request.
